# Supplementary material for: Modeling glioblastoma heterogeneity as a dynamic network of cell states
Source: Mol Syst Biol. 2021 Sep 16;17(9):e10105. doi: 10.15252/msb.202010105 (PMC8444284; doi:10.15252/msb.202010105)
Supplement: Supplementary file 6 — Source Data for Figure 5 [file MSB-17-e10105-s004.zip › Figure5A_sourcedata/GSEA_3071/nosigdb_stateIII.GseaPreranked.1622795155562/DOROSZKO_U3054MG_CLUSTER_2.html]

Details for gene set DOROSZKO\_U3054MG\_CLUSTER\_2[GSEA]

|  || Dataset | state33071 |
| Phenotype | NoPhenotypeAvailable |
| Upregulated in class | na\_neg |
| GeneSet | DOROSZKO\_U3054MG\_CLUSTER\_2 |
| Enrichment Score (ES) | -0.4663486 |
| Normalized Enrichment Score (NES) | -3.1407976 |
| Nominal p-value | 0.0 |
| FDR q-value | 0.0 |
| FWER p-Value | 0.0 |
Table: GSEA Results Summary

  

Fig 1: Enrichment plot: DOROSZKO\_U3054MG\_CLUSTER\_2      
 Profile of the Running ES Score & Positions of GeneSet Members on the Rank Ordered List

  

| PROBE | GENE SYMBOL | GENE\_TITLE | RANK IN GENE LIST | RANK METRIC SCORE | RUNNING ES | CORE ENRICHMENT || 1 | SPATS2L |  |  | 184 | 0.351 | -0.0450 | No |
| 2 | CYTOR |  |  | 258 | 0.306 | -0.0609 | No |
| 3 | AL022323.4 |  |  | 509 | -0.255 | -0.1245 | No |
| 4 | PRICKLE1 |  |  | 532 | -0.256 | -0.1274 | No |
| 5 | GLIS2 |  |  | 554 | -0.257 | -0.1301 | No |
| 6 | TLE1 |  |  | 555 | -0.257 | -0.1271 | No |
| 7 | DNM1 |  |  | 576 | -0.258 | -0.1294 | No |
| 8 | ATP6V0A1 |  |  | 581 | -0.259 | -0.1275 | No |
| 9 | FLRT2 |  |  | 676 | -0.263 | -0.1495 | No |
| 10 | CLDN1 |  |  | 692 | -0.264 | -0.1505 | No |
| 11 | SHROOM3 |  |  | 720 | -0.266 | -0.1546 | No |
| 12 | CRABP2 |  |  | 739 | -0.266 | -0.1563 | No |
| 13 | PLXDC2 |  |  | 759 | -0.267 | -0.1583 | No |
| 14 | PLAGL1 |  |  | 783 | -0.268 | -0.1613 | No |
| 15 | FRMD4A |  |  | 819 | -0.270 | -0.1675 | No |
| 16 | PLPPR5 |  |  | 824 | -0.271 | -0.1655 | No |
| 17 | SERTAD4 |  |  | 849 | -0.272 | -0.1687 | No |
| 18 | ACTN1 |  |  | 879 | -0.273 | -0.1733 | No |
| 19 | ID3 |  |  | 880 | -0.273 | -0.1701 | No |
| 20 | CDC42EP5 |  |  | 888 | -0.274 | -0.1688 | No |
| 21 | ODF2L |  |  | 894 | -0.274 | -0.1670 | No |
| 22 | NBEA |  |  | 913 | -0.275 | -0.1686 | No |
| 23 | ASPH |  |  | 965 | -0.278 | -0.1790 | No |
| 24 | BOC |  |  | 1034 | -0.281 | -0.1939 | No |
| 25 | MAP1A |  |  | 1046 | -0.282 | -0.1936 | No |
| 26 | CPE |  |  | 1081 | -0.284 | -0.1993 | No |
| 27 | CLMP |  |  | 1085 | -0.284 | -0.1969 | No |
| 28 | EMILIN1 |  |  | 1099 | -0.285 | -0.1970 | No |
| 29 | SRPX2 |  |  | 1127 | -0.286 | -0.2009 | No |
| 30 | RAMP2 |  |  | 1137 | -0.286 | -0.2000 | No |
| 31 | TFPI |  |  | 1141 | -0.287 | -0.1975 | No |
| 32 | EFNB1 |  |  | 1198 | -0.290 | -0.2091 | No |
| 33 | TIAM1 |  |  | 1276 | -0.294 | -0.2262 | No |
| 34 | PLEKHH2 |  |  | 1282 | -0.294 | -0.2241 | No |
| 35 | CDC42EP2 |  |  | 1289 | -0.295 | -0.2223 | No |
| 36 | COL18A1 |  |  | 1302 | -0.295 | -0.2221 | No |
| 37 | SLIT2 |  |  | 1400 | -0.300 | -0.2445 | No |
| 38 | SNED1 |  |  | 1405 | -0.300 | -0.2421 | No |
| 39 | ANGPTL1 |  |  | 1442 | -0.303 | -0.2482 | No |
| 40 | TGIF1 |  |  | 1506 | -0.306 | -0.2615 | No |
| 41 | LRRN4CL |  |  | 1530 | -0.308 | -0.2640 | No |
| 42 | OLFM2 |  |  | 1548 | -0.309 | -0.2650 | No |
| 43 | KLHL13 |  |  | 1566 | -0.310 | -0.2659 | No |
| 44 | ZEB1 |  |  | 1569 | -0.310 | -0.2629 | No |
| 45 | ITGA11 |  |  | 1570 | -0.310 | -0.2593 | No |
| 46 | ANTXR1 |  |  | 1578 | -0.311 | -0.2576 | No |
| 47 | ARHGAP29 |  |  | 1614 | -0.313 | -0.2633 | No |
| 48 | CDKN1A |  |  | 1631 | -0.314 | -0.2639 | No |
| 49 | TENM2 |  |  | 1734 | -0.321 | -0.2874 | No |
| 50 | HTRA3 |  |  | 1738 | -0.321 | -0.2845 | No |
| 51 | COL12A1 |  |  | 1764 | -0.322 | -0.2874 | No |
| 52 | FGFR1 |  |  | 1770 | -0.323 | -0.2850 | No |
| 53 | LAMA2 |  |  | 1786 | -0.324 | -0.2853 | No |
| 54 | GLI2 |  |  | 1843 | -0.327 | -0.2964 | No |
| 55 | P3H1 |  |  | 1846 | -0.327 | -0.2932 | No |
| 56 | PDE5A |  |  | 1869 | -0.328 | -0.2953 | No |
| 57 | COL5A3 |  |  | 1895 | -0.329 | -0.2981 | No |
| 58 | PCDH19 |  |  | 1901 | -0.329 | -0.2957 | No |
| 59 | DAAM1 |  |  | 1915 | -0.330 | -0.2953 | No |
| 60 | MAPK8IP1 |  |  | 1949 | -0.332 | -0.3003 | No |
| 61 | SPRY4 |  |  | 1970 | -0.334 | -0.3017 | No |
| 62 | ANKH |  |  | 1978 | -0.334 | -0.2998 | No |
| 63 | CAPN5 |  |  | 1994 | -0.335 | -0.2999 | No |
| 64 | MXRA8 |  |  | 1995 | -0.335 | -0.2960 | No |
| 65 | RHOU |  |  | 2004 | -0.335 | -0.2943 | No |
| 66 | IL17RD |  |  | 2073 | -0.339 | -0.3085 | No |
| 67 | GPR162 |  |  | 2080 | -0.340 | -0.3061 | No |
| 68 | PLAC9 |  |  | 2086 | -0.341 | -0.3035 | No |
| 69 | FAM198B |  |  | 2094 | -0.341 | -0.3015 | No |
| 70 | SLC2A3 |  |  | 2117 | -0.343 | -0.3034 | No |
| 71 | MFAP2 |  |  | 2169 | -0.347 | -0.3130 | No |
| 72 | FOXS1 |  |  | 2195 | -0.349 | -0.3156 | No |
| 73 | CGNL1 |  |  | 2224 | -0.350 | -0.3190 | No |
| 74 | ECE1 |  |  | 2228 | -0.351 | -0.3157 | No |
| 75 | SERPINF1 |  |  | 2245 | -0.352 | -0.3159 | No |
| 76 | SEZ6L2 |  |  | 2251 | -0.353 | -0.3132 | No |
| 77 | TMEM59L |  |  | 2359 | -0.362 | -0.3375 | No |
| 78 | LTBP4 |  |  | 2372 | -0.363 | -0.3365 | No |
| 79 | TLE2 |  |  | 2477 | -0.372 | -0.3599 | No |
| 80 | ECM2 |  |  | 2491 | -0.373 | -0.3591 | No |
| 81 | PLK3 |  |  | 2526 | -0.375 | -0.3638 | No |
| 82 | ATP13A2 |  |  | 2584 | -0.381 | -0.3746 | No |
| 83 | ISLR |  |  | 2675 | -0.388 | -0.3941 | No |
| 84 | ID1 |  |  | 2685 | -0.389 | -0.3920 | No |
| 85 | NOTCH3 |  |  | 2759 | -0.395 | -0.4069 | No |
| 86 | KCNE4 |  |  | 2796 | -0.399 | -0.4119 | No |
| 87 | ASPN |  |  | 2821 | -0.401 | -0.4137 | No |
| 88 | ABI3BP |  |  | 2870 | -0.405 | -0.4218 | No |
| 89 | LDLR |  |  | 2880 | -0.406 | -0.4195 | No |
| 90 | LIF |  |  | 2908 | -0.409 | -0.4219 | No |
| 91 | C11orf96 |  |  | 2948 | -0.414 | -0.4276 | No |
| 92 | ARL4C |  |  | 2985 | -0.418 | -0.4323 | No |
| 93 | SCN9A |  |  | 2996 | -0.420 | -0.4301 | No |
| 94 | EMP1 |  |  | 2998 | -0.420 | -0.4256 | No |
| 95 | MRC2 |  |  | 3032 | -0.424 | -0.4295 | No |
| 96 | CCND2 |  |  | 3037 | -0.425 | -0.4256 | No |
| 97 | PPIC |  |  | 3045 | -0.425 | -0.4226 | No |
| 98 | OLFML2B |  |  | 3050 | -0.425 | -0.4187 | No |
| 99 | AC009041.2 |  |  | 3139 | -0.436 | -0.4371 | No |
| 100 | PKDCC |  |  | 3160 | -0.439 | -0.4374 | No |
| 101 | CREB3L1 |  |  | 3178 | -0.441 | -0.4368 | No |
| 102 | CD248 |  |  | 3198 | -0.442 | -0.4368 | No |
| 103 | SYT1 |  |  | 3200 | -0.442 | -0.4320 | No |
| 104 | ST8SIA4 |  |  | 3296 | -0.459 | -0.4520 | No |
| 105 | EDNRA |  |  | 3351 | -0.471 | -0.4609 | Yes |
| 106 | MEST |  |  | 3363 | -0.473 | -0.4584 | Yes |
| 107 | C1R |  |  | 3364 | -0.473 | -0.4529 | Yes |
| 108 | ABCA5 |  |  | 3377 | -0.475 | -0.4506 | Yes |
| 109 | ZDBF2 |  |  | 3415 | -0.483 | -0.4549 | Yes |
| 110 | EGFR |  |  | 3433 | -0.488 | -0.4538 | Yes |
| 111 | PLPP3 |  |  | 3439 | -0.490 | -0.4495 | Yes |
| 112 | PLXDC1 |  |  | 3472 | -0.499 | -0.4522 | Yes |
| 113 | OMD |  |  | 3481 | -0.501 | -0.4486 | Yes |
| 114 | COL21A1 |  |  | 3488 | -0.504 | -0.4444 | Yes |
| 115 | C16orf45 |  |  | 3489 | -0.504 | -0.4386 | Yes |
| 116 | OLFML3 |  |  | 3503 | -0.509 | -0.4362 | Yes |
| 117 | TRIL |  |  | 3505 | -0.510 | -0.4305 | Yes |
| 118 | ZFHX4 |  |  | 3540 | -0.524 | -0.4335 | Yes |
| 119 | SOX9 |  |  | 3560 | -0.531 | -0.4325 | Yes |
| 120 | NRP1 |  |  | 3562 | -0.532 | -0.4266 | Yes |
| 121 | ATP2B1 |  |  | 3580 | -0.539 | -0.4249 | Yes |
| 122 | LRP1 |  |  | 3584 | -0.540 | -0.4195 | Yes |
| 123 | RASSF2 |  |  | 3586 | -0.541 | -0.4135 | Yes |
| 124 | LRRC17 |  |  | 3590 | -0.543 | -0.4080 | Yes |
| 125 | NES |  |  | 3596 | -0.545 | -0.4031 | Yes |
| 126 | THBS1 |  |  | 3599 | -0.547 | -0.3973 | Yes |
| 127 | SPRY1 |  |  | 3605 | -0.552 | -0.3922 | Yes |
| 128 | IGFBP7 |  |  | 3611 | -0.554 | -0.3872 | Yes |
| 129 | RGS16 |  |  | 3616 | -0.555 | -0.3818 | Yes |
| 130 | CTTNBP2 |  |  | 3627 | -0.563 | -0.3780 | Yes |
| 131 | GAD1 |  |  | 3644 | -0.574 | -0.3756 | Yes |
| 132 | SLC5A3 |  |  | 3647 | -0.575 | -0.3695 | Yes |
| 133 | CFH |  |  | 3653 | -0.582 | -0.3641 | Yes |
| 134 | AHNAK |  |  | 3660 | -0.584 | -0.3590 | Yes |
| 135 | HGF |  |  | 3662 | -0.585 | -0.3525 | Yes |
| 136 | COL4A2 |  |  | 3664 | -0.586 | -0.3460 | Yes |
| 137 | PDGFRB |  |  | 3678 | -0.598 | -0.3426 | Yes |
| 138 | FRMD4B |  |  | 3695 | -0.611 | -0.3398 | Yes |
| 139 | TCF4 |  |  | 3696 | -0.612 | -0.3327 | Yes |
| 140 | BMP7 |  |  | 3698 | -0.613 | -0.3259 | Yes |
| 141 | COLEC12 |  |  | 3699 | -0.613 | -0.3188 | Yes |
| 142 | CDK6 |  |  | 3700 | -0.614 | -0.3118 | Yes |
| 143 | ITPR1 |  |  | 3706 | -0.623 | -0.3059 | Yes |
| 144 | DCN |  |  | 3723 | -0.633 | -0.3029 | Yes |
| 145 | COL5A2 |  |  | 3726 | -0.636 | -0.2960 | Yes |
| 146 | VCAN |  |  | 3741 | -0.660 | -0.2922 | Yes |
| 147 | ADAMTS9 |  |  | 3761 | -0.687 | -0.2893 | Yes |
| 148 | SULF2 |  |  | 3776 | -0.708 | -0.2848 | Yes |
| 149 | PCOLCE |  |  | 3777 | -0.708 | -0.2767 | Yes |
| 150 | CDH11 |  |  | 3789 | -0.721 | -0.2713 | Yes |
| 151 | FBN1 |  |  | 3790 | -0.723 | -0.2629 | Yes |
| 152 | INHBA |  |  | 3801 | -0.746 | -0.2570 | Yes |
| 153 | COL8A1 |  |  | 3805 | -0.752 | -0.2491 | Yes |
| 154 | SSPN |  |  | 3808 | -0.756 | -0.2409 | Yes |
| 155 | BGN |  |  | 3812 | -0.761 | -0.2329 | Yes |
| 156 | COL5A1 |  |  | 3815 | -0.764 | -0.2246 | Yes |
| 157 | COL11A1 |  |  | 3828 | -0.788 | -0.2187 | Yes |
| 158 | COL4A1 |  |  | 3829 | -0.791 | -0.2096 | Yes |
| 159 | CARMN |  |  | 3831 | -0.794 | -0.2007 | Yes |
| 160 | CLU |  |  | 3846 | -0.827 | -0.1949 | Yes |
| 161 | FN1 |  |  | 3850 | -0.842 | -0.1860 | Yes |
| 162 | LUM |  |  | 3861 | -0.870 | -0.1786 | Yes |
| 163 | SCRG1 |  |  | 3869 | -0.904 | -0.1700 | Yes |
| 164 | NGFR |  |  | 3872 | -0.937 | -0.1597 | Yes |
| 165 | PTCH1 |  |  | 3883 | -1.016 | -0.1507 | Yes |
| 166 | POSTN |  |  | 3885 | -1.025 | -0.1391 | Yes |
| 167 | CXCL14 |  |  | 3892 | -1.060 | -0.1285 | Yes |
| 168 | COL6A3 |  |  | 3900 | -1.171 | -0.1168 | Yes |
| 169 | CTGF |  |  | 3901 | -1.185 | -0.1031 | Yes |
| 170 | COL1A1 |  |  | 3902 | -1.191 | -0.0894 | Yes |
| 171 | IGFBP3 |  |  | 3903 | -1.199 | -0.0756 | Yes |
| 172 | NRP2 |  |  | 3905 | -1.241 | -0.0615 | Yes |
| 173 | COL1A2 |  |  | 3906 | -1.278 | -0.0467 | Yes |
| 174 | DCX |  |  | 3911 | -1.320 | -0.0326 | Yes |
| 175 | COL3A1 |  |  | 3918 | -1.418 | -0.0178 | Yes |
| 176 | PDGFRA |  |  | 3928 | -1.773 | 0.0003 | Yes |
Table: GSEA details [plain text format]

  

Fig 2: DOROSZKO\_U3054MG\_CLUSTER\_2: Random ES distribution      
 Gene set null distribution of ES for **DOROSZKO\_U3054MG\_CLUSTER\_2**
